# P-1941. Risk Factors for Developing Candidemia after Gastrointestinal Perforation and/or Ischemia and Its Outcomes: A Matched Case-Control Study

**DOI:** 10.1093/ofid/ofaf695.2109

**Published:** 2026-01-11

**Authors:** Myeongji Kim, Nischal Ranganath, Sofia Molina Garcia, Kemar O Barrett, Ryan W W Stevens, Allison LeMahieu, Veljko Strajina, Aditya Shah

**Affiliations:** Mayo Clinic, Rochester, MN; Mayo Clinic, Rochester, MN; Mayo Clinic, Rochester, MN; Mayo Clinic, Rochester, MN, Rochester, MN; Mayo Clinic, Rochester, MN; Mayo Clinic Rochester, Rochester, Minnesota; Mayo Clinic Rochester, Rochester, Minnesota; Mayo Clinic, Rochester, MN

## Abstract

**Background:**

Candidemia can occur as a consequence of gastrointestinal (GI) injury. The mortality of candidemia is high, ranging 30-60%, even with treatment with antifungal agents. In this retrospective matched case-control study, we aimed to elucidate the medical and surgical risk factors of developing candidemia in patients who received emergency surgery for GI ischemia and/or perforation, and compare clinical outcomes in these patients with or without candidemia.

**Figure:**
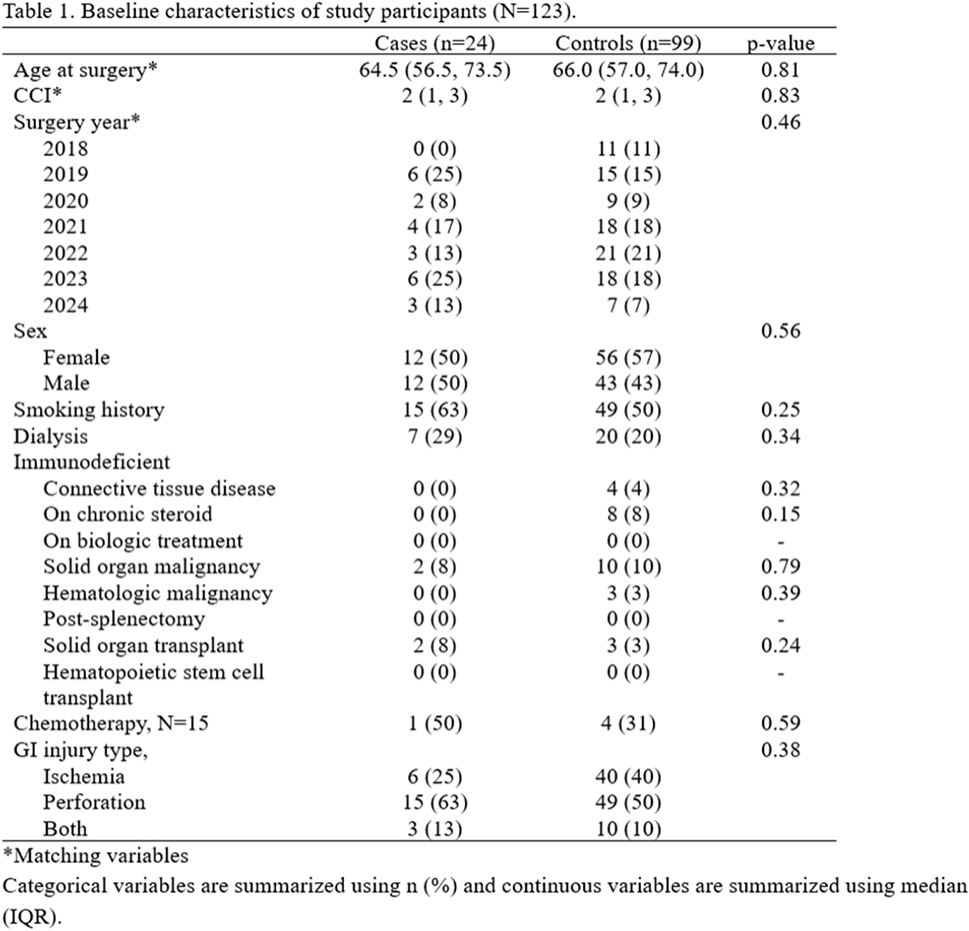

**Figure:**
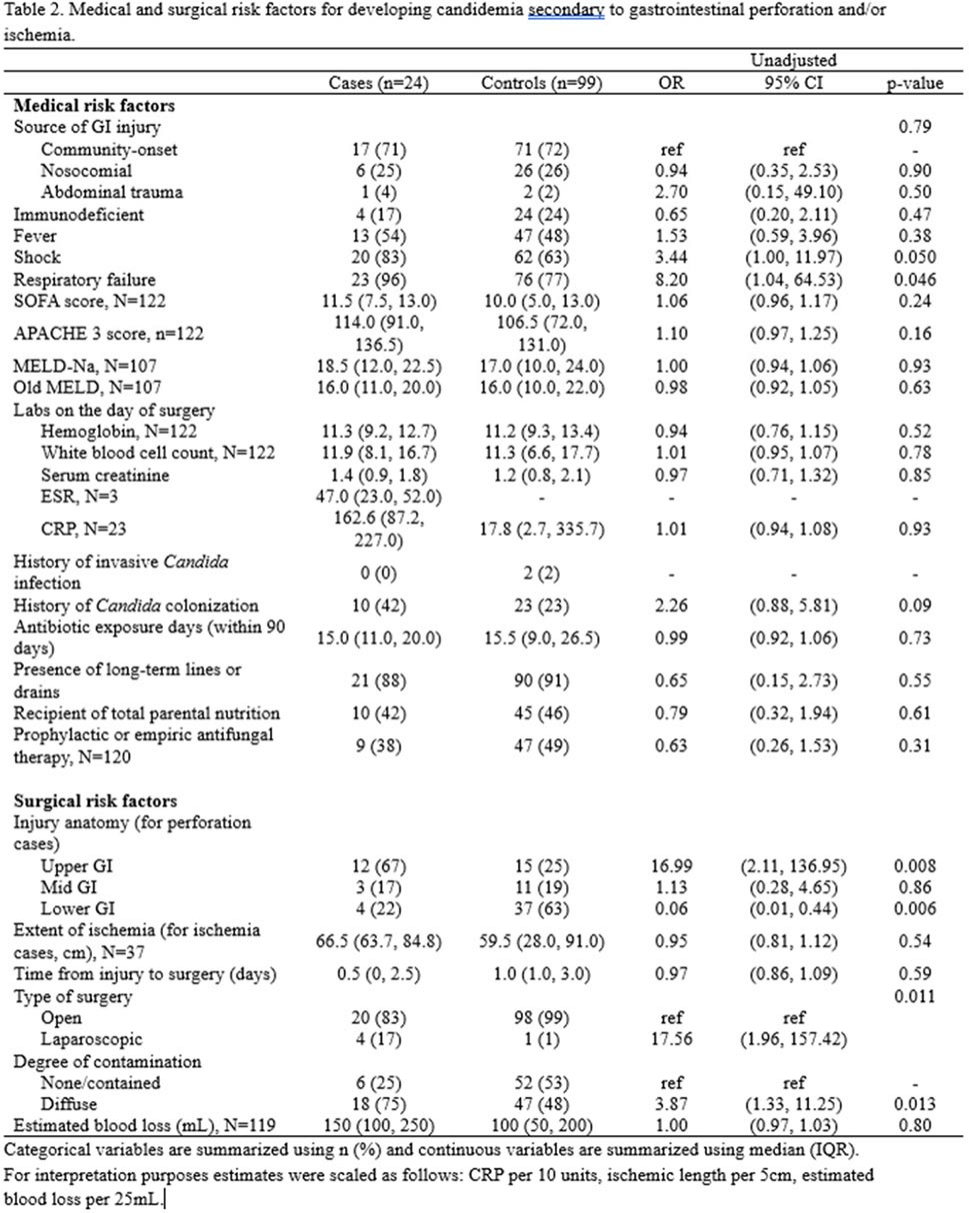

**Methods:**

All patients ≥ 18 years of age who had emergency GI surgery at Mayo Clinic Rochester, MN, for the indication of GI ischemia and/or perforation between 01/01/2018 to 07/31/2024 were included. Cases were defined as patients who developed candidemia during index admission and had no alternative sources of candidemia. Controls and cases were matched 4:1 on age (± 5 years), Charlson Comorbidity Index (± 3), and year of surgery (± 2 years). The relationships between risk factors of interest and case/control were assessed using conditional logistic regression.

**Figure:**
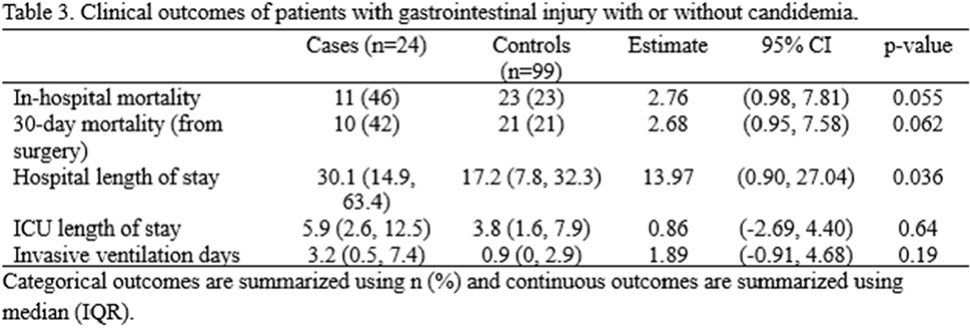

**Results:**

Among medical risk factors, any shock requiring vasopressor (OR = 3.44, 95% CI 1.00, 11.97) or respiratory failure (OR = 8.20, 95% CI 1.04, 64.53) during hospitalization were associated with increased risk of candidemia. Among surgical risk factors, perforation in the upper GI tract was strongly associated with development of candidemia (OR = 16.99, 95% CI 2.11, 136.95) and perforation in the lower GI tract were less likely to lead to candidemia (OR = 0.06, 95% CI 0.01, 0.44). The patients who had diffuse intraabdominal contamination were more likely to develop candidemia compared to those who had no contamination or contained contamination (OR = 3.87, 95% CI 1.33, 11.25). The patients with candidemia showed signal towards higher in-hospital and 30-day mortality, and had significantly longer stay in the hospital.

**Conclusion:**

Candidemia was more frequently identified in patients with shock, respiratory failure, upper GI perforation, and intra-abdominal contamination, and contributed to significant morbidity and mortality. Further analysis will include building a multivariate logistic regression model to implement prospective trial for early initiation of antifungal therapy for high-risk population.

**Disclosures:**

All Authors: No reported disclosures

